# Dementia: timely diagnosis and early intervention

**DOI:** 10.1136/bmj.h3029

**Published:** 2015-06-16

**Authors:** Louise Robinson, Eugene Tang, John-Paul Taylor

**Affiliations:** 1 Institute of Health and Society, Newcastle University, Newcastle upon Tyne, NE2 4AX, UK; 2 Institute of Neuroscience, Newcastle University, Newcastle upon Tyne, UK

**The bottom line**

- Dementia is a major global health problem; in the absence of a cure there is increasing focus on risk reduction, timely diagnosis, and early intervention
- Primary and secondary care doctors play complementary roles in dementia diagnosis; differential diagnoses include cognitive impairment due to normal ageing and depression
- Cost effective drug (acetylcholinesterase inhibitors) and non-drug interventions such as cognitive stimulation therapy exist that help to delay cognitive deterioration and improve quality of life; information provision and practical support are also important
- Discussions about a person’s wishes for future care should occur at an early stage of illness while the person has mental capacity
- Family carers of people with dementia are at high risk of physical and mental illness as a consequence of caring and they require equal attention and support

Dementia describes a clinical syndrome that encompasses difficulties in memory, language, and behaviour that leads to impairments in activities of daily living. Alzheimer’s disease is the most common subtype of dementia, followed by vascular dementia, mixed dementia, and dementia with Lewy bodies. Because the global population is rapidly ageing, dementia has become a concern worldwide^1^; the illness places considerable burden on individuals and their families and also on health and social care provision.

**Sources and selection criteria**

We searched for articles through Medline, PubMed, and the Cochrane database of systematic reviews from January 2006 to December 2014—the period after publication of the current UK national dementia guidance^9^—using the search terms “dementia”, “Alzheimer’s”, “carer”, and “caregiver”. Additional searches were carried out for specific subsections—for example, “pharmacologic treatment” and “non-pharmacologic interventions/strategies/treatment”. Where possible, we focused on systematic reviews, meta-analyses, and high quality randomised controlled trials. We included only articles in English and excluded those published in non-peer reviewed journals. Recommendations in this review are derived from the most recent international and UK national guidance^9^ on evidence based practice in dementia care and the authors’ interpretation of the included evidence.

By 2050 an estimated 135 million people worldwide will have dementia. In 2010 the global cost of dementia care was estimated at $604bn (£396bn; €548bn) and estimated to increase to $1tr by 2030.^1^ Of all chronic diseases, dementia is one of the most important contributors to dependence and disability.^2^ ^3^ In the absence of a cure, a professional belief that nothing can be done has contributed to delays in diagnosis.^4^ However, increasing evidence showing that dementia may be preventable^1^ ^5^ has led to an international focus on earlier diagnosis and intervention.^6^ This review aims to summarise current evidence and best practice in the diagnosis and early intervention in dementia care.

**Patient and public involvement**

Patient and public involvement in this clinical review has been achieved through several processes: the inclusion of patients and the public in the groups responsible for developing the national guidelines referenced in this review; liaising with patient and public representatives from the National Institute of Health Research Dementia and Neurodegenerative Diseases Research Network who contributed to systematic reviews included in this review^4^ ^9^; and asking the UK Alzheimer’s Society to comment on the final draft of the paper and provide up to date information resources for patients and carers.

## Why is timely diagnosis important?

In some countries the introduction of a national dementia strategy has led to greater emphasis on earlier diagnosis, although population based screening is not recommended as dementia does not fulfil the criteria of a condition suitable for screening.^7^ With evidence from large longitudinal cohort studies showing that the prevalence of dementia is declining globally, there is now greater emphasis on prevention and risk reduction.^1^ ^5^ In England, policy has rightly or wrongly influenced the introduction of case finding in high risk groups—including people over 75 years of age, as age is the strongest risk factor for dementia—and those with high vascular risk, Parkinson’s disease, and learning disabilities.^8^ The policy comprises proactive memory assessment of people in both primary care and acute hospital settings who may not have symptoms; however, there is little evidence that such initiatives, which inevitably lead to increased referrals to specialist services, are cost effective and whether they are distressing to patients.^4^ ^6^

## How can clinicians recognise dementia?

Diagnosing dementia can be difficult owing to its insidious onset, symptoms resembling “normal ageing” memory loss, and a diversity of other presenting symptoms—for example, difficulty in finding words or making decisions.^10^ An individual’s ability to accommodate, compensate, or even deny his or her symptoms in the early stages should also be considered. The individual’s family may also have noticed difficulties in communication and personality or mood changes; family concern is of particular importance.^9^ Increasing frequency of patients’ visits to their general practice, missed appointments, or confusion over drugs may also be warning signs.^8^

Diagnosis of subtype is important given differences in management, disease course, and outcomes for different dementias; awareness of early symptoms in less common dementias can assist generalists in deciding to which specialist services patients are referred (box 1). Duration over which symptoms have developed is also important, with Alzheimer’s disease tending to have a more insidious onset than vascular dementia.

**Box 1 Examples of less common dementias and their early presenting symptoms**

**Vascular dementia**

- Wide range of signs and symptoms depending on extent, location, and severity of the cerebrovascular disease
- Symptoms can develop abruptly after a stroke or more insidiously with small vessel disease
- Memory loss can be a feature but typically is less noticeable than in Alzheimer’s disease. Language, information processing, decision making, and visuospatial deficits can also be found
- Mood changes and apathy are common symptoms; can co-occur with Alzheimer’s disease and this is termed mixed dementia

**Frontotemporal dementias**

- More common in younger age groups (50-60 years)
- The most common clinical type is behavioural variant frontotemporal dementia, with changes in personality and behaviour. Disinhibition and impulsiveness can be features. Memory function is typically intact early on

**Dementia with Lewy bodies**

- Complex visual hallucinations are a key feature. In the early stages they may only occur during periods of physical stress (for example, infections) or at night time and may be followed by more subtle visuoperceptual symptoms—for example, illusions
- Parkinsonism (tremor, slowed movements, postural instability, shuffling gait) is also a feature. Tremor may be less evident, but people with early dementia with Lewy bodies may be slower in movements and more prone to falls
- Fluctuations or noticeable variations in cognitive function can occur and can be difficult to separate from delirium
- Autonomic symptoms may occur—for example, postural hypotension
- Sleep disturbances such as rapid eye movement sleep behaviour disorder (shouting out or moving while asleep) can occur many years before the onset of dementia

**Parkinson’s disease with dementia**

- As many as 80% of patients with Parkinson’s develop dementia
- Symptoms are similar to those of dementia with Lewy bodies, although motor Parkinson’s symptoms typically predate cognitive and psychiatric symptoms by more than a year

**Posterior cortical atrophy**

- A less common form of Alzheimer’s disease, which tends to affect younger people (50s and 60s)
- Visual agnosias (difficulties with recognising faces, objects, or perceiving more than one object at a time), apraxias (motor planning difficulties), acalculia (difficulty with calculation), and alexia (difficulty reading) are symptoms
- Memory typically preserved early on

**Other uncommon to rare causes of dementia**

- Alcohol related dementia, Creutzfeldt-Jakob disease, HIV related cognitive impairment, Huntington’s chorea, corticobasal syndrome, movement related dementias (for example, progressive supranuclear palsy), multiple sclerosis, Niemann-Pick disease type C, normal pressure hydrocephalus

## How is dementia diagnosed?

### The role of primary care

General practitioners are often the first point of contact for patients who are worried that they may have dementia. The role of primary care is to exclude a potentially treatable illness or reversible cause of the “dementia”—for example, depression, vitamin B_12_ deficiency, or thyroid disturbance; refer for specialist assessment, especially those with unusual symptoms (neurological, psychiatric, or behavioural changes) or those with major risk factors (for example, important medical comorbidities, psychosocial problems, harm to self); and ensure patients who have mild cognitive impairment (objective cognitive loss not affecting function and daily living activities) are followed up in primary care, and, if their symptoms become more severe, re-referred for specialist assessment.

Initial assessment should include a careful history from both the patient and the main carer, with particular emphasis on disturbance of cognitive function and activities of daily living. A physical examination should be undertaken to look for any focal neurological signs and exclude any visual or auditory problems. Baseline investigations and a brief cognitive assessment, using one of the many tools available (box 2), should also be carried out before referral to secondary care.^9^

**Box 2 Investigations and brief cognitive assessment tools for dementia in primary care**

**Blood tests**

Blood tests that should be ordered are: full blood count, erythrocyte sedimentation rate, urea and electrolytes, thyroid function, vitamin B_12_, and folate. Midstream specimen of urine, chest radiography, and electrocardiography may also be needed where clinically appropriate

**Brief cognitive assessment tools**

**General practitioner assessment of cognition^11^**

Takes no longer than five minutes to administer and comprises two components: a six item cognitive assessment with the patient and an informant questionnaire (if the cognitive assessment score is equivocal: 5-8 inclusive). Scores >8 are deemed to represent cognitive impairment and <5 intact cognition. Sensitivity 82-85%; specificity 83-86%^12^

**6 item cognitive impairment test^13^**

Takes 3-4 minutes to perform and consists of six questions on orientation and memory, although the test may be susceptible to influences of language and education. Scores of 0-7 are considered normal and ≥8 suggest cognitive impairment. Sensitivity 78.5-83%; specificity 77-100%^12^

**Mini-cog assessment instrument^14^**

Takes 2-4 minutes to complete and consists of two components, a three item recall and the clock drawing test. Cognitive impairment is considered to be present if people are unable to recall any of the three items or if they recall only one or two items and draw an abnormal clock. Sensitivity 76-99%; specificity 89-96%^12^

**Memory impairment screen^15^**

Takes around four minutes to complete and is a brief four item delayed free recall and cued recall memory impairment test. A score of ≤4 indicates possible dementia. Sensitivity 74-86%; specificity 96-97%^12^

The mini-mental state examination^16^ has traditionally been recommended as the brief cognitive assessment tool of choice, although copyright restrictions are influencing its use in practice. The tools listed in box 2 have been found to be as clinically and psychometrically robust as the mini-mental state examination^17^; a clock drawing test may be added to the assessment if it is not already incorporated into the tool.^18^ The Addenbrooke’s cognitive examination,^19^ especially the revised version, has superior diagnostic accuracy to the mini-mental state examination but takes about 25 minutes to complete and has better accuracy in moderate to high prevalence settings.^20^ No one brief cognitive assessment tool is more accurate than another and all are inadequate for assessing early or subtle changes, with scores affected by factors such as education. Mini-mental state examination scores are used to indicate the severity of Alzheimer’s disease: mild, scores 21-26; moderate, scores 10-20; moderately severe, scores 10-14; severe, scores less than 10.

Depression masquerading as dementia is probably the most common differential diagnosis and should always be considered; however, they can coexist and depression may precede dementia. If suspected, a trial of antidepressants may be indicated, with reassessment of the individual’s capabilities and cognitive function 6-8 weeks later.

### The role of secondary care

Primary care is increasingly taking on a greater role in both the assessment and the long term care of people with dementia; one multicentre randomised controlled trial found no evidence that specialist memory clinics were more effective than general practice services in providing post-diagnostic support.^21^ Secondary services have an important role in defining the dementia subtype, dealing with more complex cases, and stratifying which patients with mild cognitive impairment are at greatest risk of developing dementia and most in need of follow-up.

## What are the roles of imaging and other investigations?

Imaging, in particular structural scanning (computed tomography or magnetic resonance imaging), is recommended as part of the investigations of people with suspected dementia in UK,^9^ European,^22^ and US guidelines.^23^ Imaging is now also embedded in several modern diagnostic criteria for different dementias, including Alzheimer’s disease and dementia with Lewy bodies.^24^ ^25^ ^26^ In modern dementia imaging there is now less focus on “excluding” reversible causes of dementia (for example, tumours) and more on determination of subtype. Structural imaging, particularly magnetic resonance imaging, can also help clarify whether a vascular disease is contributing to the cognitive impairment and thus whether strict adherence to treatment guidance for vascular risks is warranted.

In the United Kingdom, functional neuroimaging, including hexamethylpropyleneamine oxime (HMPAO) single photon emission computed tomography (SPECT) and [18F]fluoro-2-deoxy-D-glucose positron emission tomography (FDG-PET), is available but usually used as a second line approach to assist with subtype diagnoses, particularly where the diagnosis is in doubt. Dopaminergic iodine-123-radiolabelled 2β-carbomethoxy-3β-(4-iodophenyl)-N-(3-fluoropropyl) nortropane (FP-CIT) SPECT imaging is licensed in Europe and in a number of other countries for the diagnosis of dementia with Lewy bodies and may also be helpful where the clinical diagnosis of dementia with Lewy bodies is not clear.^27^

## What new investigations are emerging in the diagnosis of dementia?

Cerebrospinal fluid sampling is used to exclude inflammatory, infective, and malignancy related causes of dementia and is typically recommended in individuals with rapid cognitive decline, unusual or neurological presentations, or cognitive impairment at less than 55 years of age.^28^ More recently there has been a focus on developing cerebrospinal fluid based markers, such as β amyloid and tau, for changes in Alzheimer’s disease that can predate the onset of the dementia, the so called prodromal phase of Alzheimer’s disease. Although such markers have been incorporated into recent diagnostic criteria for Alzheimer’s disease,^25^ ^26^ whether they are effective at predicting those who will develop dementia^29^ and, more importantly, practically acceptable, makes their widespread clinical use challenging at present.

It is now possible to directly image amyloid in the brain using several positron emission tomography radiotracers, and this imaging technique may have a future role clinically in predicting which people with mild cognitive impairment will develop Alzheimer’s disease. However there is still major heterogeneity in how these scans are interpreted. For example, a recent meta-analysis found that although amyloid imaging has high sensitivity (83-100%) in detecting people with mild cognitive impairment who convert to Alzheimer’s disease related dementia, diagnostic specificities varied considerably between studies (46-88%).^30^

## What constitutes best practice in early intervention?

### Discussing the diagnosis: saying the “D” word

Health professionals can be reluctant to speak openly and honestly with patients and their families about dementia, with some refraining from using the “D” word.^28^ Although initially discussing the diagnosis may be distressing, evidence suggests most people prefer to know if they have dementia in order to access appropriate support and treatment and to plan for the future.^4^ ^31^

## What options are available after diagnosis?

### Drug interventions

Clinically and cost effective drugs for dementia are available; the emphasis is to improve or maintain function after neuronal damage rather than to alter the underlying pathogenesis leading to the dementia syndrome. Two classes of drugs are currently recommended for symptomatic (Alzheimer’s disease and mixed) dementia^6^ ^32^: acetylcholinesterase inhibitors donepezil, galantamine, and rivastigmine, and N-methyl-D-aspartic acid receptor antagonists such as memantine. At present, acetylcholinesterase inhibitors are the only recommended options to manage mild to moderate Alzheimer’s disease and there is no evidence that one is more efficacious than another^33^; notwithstanding, a large randomised controlled trial has recently shown that continued treatment with donepezil is associated with cognitive benefits in moderate to severe dementia.^34^ Memantine has been approved for people with moderate to severe Alzheimer’s disease or those with intolerance to acetylcholinesterase inhibitors; it has also been used in mild Alzheimer’s disease but the evidence for this is currently lacking despite its frequent off-label use.^35^

### Non-drug approaches

The evidence base is steadily increasing for non-drug interventions in dementia care, although further research in many areas is still needed.^6^ In a large systematic review evaluating both drug and non-drug interventions in dementia care, cognitive stimulation therapy was found to be as clinically and cost effective as the acetylcholinesterase inhibitors^36^; reminiscence therapy is also recommended in national guidelines.^9^ However, the evidence base for innovative service provision such as case management, whereby a case manager, usually a nurse or social worker acts as the main care coordinator between key stakeholders, including primary and secondary care, is mixed.^6^ ^36^ Although the evidence base for cost effectiveness is low,^37^ specially developed assistive technology—any device or system that allows an individual to perform tasks that they would otherwise be unable to do, or increases the ease and safety with which the task can be performed—to help people with dementia is available and can be useful in relieving carer anxiety and helping people with dementia to remain living at home (www.atdementia.org.uk/).

### Information provision

People with dementia and their families require emotional and practical support to help them live as good a quality of life as they can; the family doctor is in a key position to provide ongoing support and advice once the diagnosis is confirmed.^4^ ^6^ Voluntary organisations such as Alzheimer’s International provide a wide range of information resources and practical support for people living with all types of dementia (www.alz.co.uk/). Signposting to local sources of support as well as social services and respite care are integral to the consultation. Listening to an individual patient’s difficulties and concerns and providing simple cognitive and emotional strategies in the primary care consultation are beneficial to both patients and their families.

### Discussing the future

One important area to be discussed in the earlier stages of dementia, while people still have mental capacity, is personal wishes for future care and also who should make decisions when the patients are no longer able to do so. In dementia, such discussions—termed advance care planning—have been shown to reduce inappropriate hospital admissions towards the end of life, but the evidence base is weak.^38^ ^39^ Discussions about advance care planning require both sensitivity and honesty; general practitioners or hospital specialists are well placed to undertake these discussions if they have an established relationship with the patient. After such conversations, patients can formally record their wishes in several ways, including the completion of an advance directive, or “living will” as it was previously known (box 3).

**Box 3 Outcomes of advance care planning discussions: international and national terminology**

- *Statement of wishes and preferences*—this documents an individual’s wishes for future care and is not legally binding; in the UK this is known as an advance statement
- *An advance directive for refusal of treatment (or “living will”*)—this is a statement of an individual’s refusal to receive specific medical treatment in a predefined future situation. It is legally binding and comes into effect when a person loses mental capacity. In the UK, this is known as an advance decision to refuse treatment
- *A proxy decision maker or power of attorney*—This is a legally binding document whereby an individual (“donor”) nominates another (“attorney”) to make decisions on his or her behalf should he or she lose capacity. In England, following the Mental Capacity Act, this is now known as a lasting power of attorney and there are two separate aspects to lasting power of attorney, one for an individual’s health and welfare and a second for property and financial affairs

Primary care doctors may find it difficult to assess the mental capacity of an individual with dementia; mental capacity may fluctuate with time and also with acute illness. In England, the introduction of the Mental Capacity Act in 2005 provided much needed guidance for health and social care professionals on how to undertake an assessment of capacity and to make decisions in the best interests of adults who lack the mental capacity to do so for themselves (box 4).

**Box 4 Assessment of mental capacity (as derived from UK Mental Capacity Act 2005)**

**Two stage test for determining whether an individual has mental capacity to make a specific decision**

1. Does the patient have an impairment or disturbance of function of the brain?
2. Regarding a specific decision, can the patient:
  - understand the decision to be made?
  - retain sufficient information to make an informed decision?
  - use information appropriately?
  - communicate their decision?
Practical tips for assessment of capacity:
  - Information may need to be provided in different forms
  - General practitioners may need to assess patients on several occasions—that is, if morning is the best time for them
  - Record information and the two stages described above accurately in patient notes
  - Refer to experts (old age psychiatry) if in doubt

### Caring for family carers

In the UK, two thirds of people with dementia live independently in the community, with most of their care and support provided by family and friends. Such informal carers are more likely to experience depressed mood, to report a higher care “burden,” and to have worse physical health than carers of people with other long term conditions.^40^ They may grieve as their family member loses functional and cognitive abilities, and as companionship, affection, and intimacy are affected; this is termed a living bereavement. Notwithstanding the satisfaction carers experience from caring, the support they receive and their ability to seek help when needed influence how they cope. Supporting informal carers, monitoring their health and wellbeing, and providing or referring them for additional practical and psychological support is another crucial role for general practitioners and community care services.^41^

**Tips for non-specialists**

- Occasional memory lapses are common as people get older, especially in the presence of stress, depression, and acute physical illness; review the patient after appropriate treatment has been given or a reasonable length of time has elapsed
- If you suspect dementia, take a history from both the patient and the main family carer; the latter’s suspicions are often correct
- Be aware that certain groups of people are at greater risk of developing dementia—for example, those who have had a stroke and those with Parkinson’s disease
- Early identification of modifiable risk factors for dementia may reduce the numbers of people developing dementia in later life
- Effective and useful treatments exist for people with dementia; have a low threshold for referring someone with suspicious symptoms for a specialist memory assessment
- Assess both the physical and the mental health of the main family carer; supporting informal carers is an important part of dementia care

**Additional educational resources**

**Resources for healthcare professionals**

Alzheimer’s Society. Assessing cognition in older people (www.alzheimers.org.uk/cognitiveassessment)—a practical toolkit for health professionals
BMJ Group resources: BMJ Learning modules. (http://learning.bmj.com/learning/module-intro/dementia-primary-care)—describes the management of dementia in primary care
BMJ Quality (http://quality.bmj.com)—four e-projects to improve quality of care in the areas of support for carers, antipsychotic drug prescribing, timely diagnosis, and palliative care

**Resources for patients and carers**

Social Care Institute for Excellence. Dementia gateway (www/scie/org.uk/dementia)—web based information and e-learning resources written by experts mainly for professional carers and supporters
Alzheimer’s Society. The dementia guide: living well after diagnosis (www.alzheimers.org.uk/dementiaguide or request copies at publications@alzheimers.org.uk)—comprehensive practical information for people with dementia and families with a recent diagnosis. Includes a free booklet, video case studies, and downloadable translations
Lewy body Society (http://lewybody.org/aboutdlb)—website of the only charity in Europe exclusively concerned with dementia with Lewy bodies
FTD Talk (www.ftd.org)—accessible updates and web information for people with frontotemporal dementia from researchers
Alzheimer’s Disease International. Help for caregivers (www.alz.co.uk/ADI-publications)—a downloadable booklet produced in collaboration with the World Health Organization: practical tips on caring for someone with dementia
Carers UK Factsheets (http://carersuk.org)—practical information for carers about topics such as benefits and getting help and support
at dementia (www.atdementia.org.uk)—a website providing information on assistive technology for people with dementia

## Notes

We thank Tim Beanland, knowledge services manager at the Alzheimer’s Society, London for advice. LR is supported by a National Institute for Health Research professorship (NIHR-RP-011-043).

Provenance and peer review: Commissioned; externally peer reviewed.

## Notes

Cite this as: *BMJ* 2015;350:h3029
